# Fetal and Infant Outcomes in the Offspring of Parents With Perinatal Mental Disorders: Earliest Influences

**DOI:** 10.3389/fpsyt.2019.00391

**Published:** 2019-06-27

**Authors:** Evin Aktar, Jin Qu, Peter J. Lawrence, Marieke S. Tollenaar, Bernet M. Elzinga, Susan M. Bögels

**Affiliations:** ^1^Clinical Psychology Unit, Department of Psychology, Leiden University, Leiden, Netherlands; ^2^Leiden Institute for Brain and Cognition, Leiden University, Leiden, Netherlands; ^3^Department of Psychology, Clarion University of Pennsylvania, Clarion, PA, United States; ^4^Centre for Innovation in Mental Health, School of Psychology, University of Southampton, Southampton, United Kingdom; ^5^Research Institute of Child Development and Education, University of Amsterdam, Amsterdam, Netherlands; ^6^Developmental Psychology, Department of Psychology, University of Amsterdam, Amsterdam, Netherlands

**Keywords:** parents, parental mental illness, prevention, intervention, infancy, pregnancy

## Abstract

Mental illness is highly prevalent and runs in families. Mental disorders are considered to enhance the risk for the development of psychopathology in the offspring. This heightened risk is related to the separate and joint effects of inherited genetic vulnerabilities for psychopathology and environmental influences. The early years of life are suggested to be a key developmental phase in the intergenerational psychopathology transmission. Available evidence supports the idea that early exposure to parental psychopathology, during the pregnancy and first postpartum year, may be related to child psychological functioning beyond the postpartum period, up to adulthood years. This not only highlights the importance of intervening early to break the chain of intergenerational transmission of psychopathology but also raises the question of whether early interventions targeting parental mental disorders in this period may alleviate these prolonged adverse effects in the infant offspring. The current article focuses on the specific risk of psychopathology conveyed from mentally ill parents to the offspring during the pregnancy and first postpartum year. We first present a summary of the available evidence on the associations of parental perinatal mental illness with infant psychological outcomes at the behavioral, biological, and neurophysiological levels. Next, we address the effects of early interventions and discuss whether these may mitigate the early intergenerational transmission of risk for psychopathology. The summarized evidence supports the idea that psychopathology-related changes in parents’ behavior and physiology in the perinatal period are related to behavioral, biological, and neurophysiological correlates of infant psychological functioning in this period. These alterations may constitute risk for later development of child and/or adult forms of psychopathology and thus for intergenerational transmission. Targeting psychopathology or mother-infant interactions in isolation in the postnatal period may not be sufficient to improve outcomes, whereas interventions targeting both maternal psychopathology and mother-infant interactions seem promising in alleviating the risk of early transmission.

## Introduction

The transition to parenthood is a major life event that brings profound and lasting changes in new parents’ relationships and personal identities as well as in the structure and organization of daily life. Becoming parents can be experienced as a highly rewarding but also a highly demanding task (1). The responsibilities of parenthood during the first year where infants fully depend on the caregivers can be stressful especially for parents with (predispositions for) psychopathology. This is why early parenthood is considered to be a period of vulnerability for the new onset and/or relapse of psychopathology in parents.

Among different types of psychopathology that manifest perinatally, the highest incidence rates have been reported for depression. The prevalences of pregnancy and postpartum depression range between 13% (2, 3) and 25% for mothers (4) and between 8.4% (5) and 10% for fathers (4). Anxiety disorders are also highly prevalent and commonly manifest comorbid with depression (6, 7), with incidence rates between 10% and 18% for mothers (8–10) and 5% to 10% for fathers (11, 12) during the perinatal period. Although relatively less prevalent, psychosis (13) and birth-related posttraumatic stress disorder (14) may specifically manifest following birth. Earlier research on perinatal psychopathology has almost exclusively focused on the most prevalent (i.e., depression) and the most severe (i.e., psychosis) forms of psychopathology in mothers (15, 16), whereas the presence of other mental disorders in this period have only recently been acknowledged (4, 17–19). Moreover, fathers have only recently been incorporated into the studies of perinatal mental illness. Psychopathology often co-occurs in new mothers and fathers, reflecting the influences of assortive mating (20) and the effects of living with a partner with a mental illness. The presence of psychopathology in both parents may multiply the risk of transmitting mental illness to offspring (4, 12, 21, 22). Hence, a better understanding of paternal influences, alongside and in interaction with maternal influences is of paramount importance.

The variability in the prevalence estimates across studies of perinatal mental illness in parents is partly explained by other risk factors, for example, socioeconomic disadvantages, unplanned pregnancies, low empathy, and social support from the partner and/or environment (23, 24). Furthermore, the link between parental mental illness and offspring psychopathology may mediate the effect of other disadvantages that are known to be intergenerationally transmitted, such as childhood emotional abuse and neglect in parents (25). Childhood maltreatment constitutes a lifelong risk for depression (26, 27) that may specifically manifest during transition to parenthood (28–30). Depression in parents with these adverse childhood experiences increases the risk of child maltreatment, and infants’ postnatal exposure to maternal depression and maltreatment, in turn, multiplies the risk of psychopathology in the offspring.

There is substantial continuity in perinatal psychopathology (31–33), the strongest risk factor for psychopathology during the postnatal period is prenatal psychopathology. Estimates are that over 50% of the mental disorders reported in the postnatal period are relapses of prenatal psychopathology (2, 19). Despite a clear accumulation of risk on parents with earlier mental disorder, psychopathology in new parents goes undetected almost in half of the cases (34, 35). Undetected and untreated psychopathology in this period can take a chronic form, especially in case of a previous history of mental illness. The impact on the child of chronic and recurrent psychopathology in parents, extending beyond the prepartum and postpartum period, would be more profound and present a more pronounced risk for intergenerational transmission of psychopathology (36, 37).

Along with the studies focusing on the prevalence of mental illness during the pregnancy and postnatal period in community samples, a related line of research focuses on the needs and experiences of individuals with chronic and severe mental disorders (such as psychotic disorders) in the reproductive age (38, 39). A meta-synthesis of the qualitative evidence on the early experiences of mothers with severe mental illness reveals several challenges on the way to parenthood (40). At the core, these seem to result from the inherent conflict between the desire to be a good mother as defined by society and the limitations coming from living with a severe mental illness. Mothers experience guilt over their maternal abilities and over the risk of transmitting mental illness to their child. Moreover, the stigma of mental illness seems to be enhanced in the case of motherhood, making mothers less likely to seek help for the challenges they encounter and more likely to end up feeling isolated in this period (40). Early experiences of parenthood in men with chronic or severe mental disorders still remain to be incorporated into this line of research.

Taken together, available evidence on perinatal psychopathology and on the experiences of motherhood in women with severe mental disorders clearly illustrates that the transition to parenthood is a vulnerable phase on the side of parents. The vulnerability on the side of infants, in turn, is related to the tremendous changes and fast-paced development that takes place in the infant brain in this period (41, 42). These changes are highly dependent on infants’ environmental experiences. Early experiences have the power to impact on the ongoing brain development either by altering or by moderating the developing function or structure of the infant brain (43). This sensitivity to environmental input by newborns and new parents explains why early environmental adversity including parental psychopathology may have an especially pronounced impact on infants’ development in the early years of life (44, 45). For example, prenatal exposure to parental stress in the context of depression and anxiety is linked with changes in the development of infant hypothalamic–pituitary–adrenal (HPA) axis (46, 47), and postnatal exposure to psychopathology is suggested to influence the development of the key emotional brain systems for adult emotion processing, which become functional at around the first year of life (48–50).

Studies on the relationship between mental illness in parents and psychological functioning in the offspring have been categorized broadly into a micro versus macro perspective (44). Within the context of the perinatal period, the micro perspective focuses on the immediate associations of parental prenatal and/or postnatal mental illness with infant development, with a specific focus on aspects of early psychological functioning that may play a role in later psychopathology. The macro perspective, in turn, focuses on the longitudinal measurement of psychopathology in the offspring of parents with perinatal mental disorder over time intervals that extend from infancy up till adulthood.

Available evidence from the macro perspective reveals that parental psychopathology in the perinatal period may be related to child functioning beyond early years. At least in some cases, this link holds after taking into account later psychopathology in parents. This would reflect the specific influence of both genetically inherited dispositions for psychopathology and early environmental influences related to being exposed to a parent with mental illness in utero and in early life. To illustrate with the most studied mental disorder, i.e., maternal depression, studies reveal a significant link between exposure to maternal depression during pregnancy and the first postpartum year, and psychological functioning in the offspring from infancy to adulthood years. For example, infants of mothers with prenatal depression show more internalizing and externalizing problems at 1 year of age (51). Children of mothers with postnatal depression show more behavioral problems at the age of 2 (52), and of 5 and beyond (53, 54), along with a higher (up to fourfold to fivefold) risk of mental disorders such as depression and anxiety at 11 (55), 13 (56), and 16 years of age (57). There is also some evidence revealing similar effects of fathers’ depression (58, 59), and parents’ anxiety disorders in this period on child outcomes (60–62). Other studies have revealed more modest estimates of this link and have highlighted the importance of incorporating the chronicity of parental mental illness and other risk factors into this line of research (55, 63–67). Thus, further research is needed before we can reach firm conclusions about distinct associations of parental mental disorder at the perinatal period with later psychopathology in the offspring, whereas the evidence accrued so far from the macro perspective points to a link between offsprings’ early exposure to parental psychopathology and later development of psychopathology. This highlights the importance of intervening early to break the chain of intergenerational transmission of psychopathology. As suggested by the antenatal investment hypothesis, the earlier the interventions are, the higher the returns would be in terms of economic and social benefits (68).

The findings from the macro perspective illustrate the need to observe early processes that are potential precursors to psychopathology in the offspring of mentally ill parents over the course of development from a micro perspective. The aims of this current review focusing on the immediate infant psychological outcomes from the micro perspective are twofold. The first is to gain insight in the effects of parental perinatal mental illness on early functioning by providing an overview of the associations of parental mental illness with infant psychological outcomes at the behavioral (see the section Behavioral Pathways: The Relationship Between Parental Perinatal Mental Disorder and Early Indices of Infant Psychobehavioral Functioning), biological (see the section Biological Pathways: The Links Between Parental Prenatal Mental Disorder and Early Indices of Infant Psychobiological Functioning), and neurophysiological levels (see the section Neurophysiological Pathways: The Links Between Parental Perinatal Mental Disorder and Early Neurophysiological Indices of Infant Psychological Functioning). The second aim is to answer the question of whether early interventions may mitigate the early intergenerational transmission of risk for psychopathology (see the section Effect of Early Interventions on Parent and Infant Outcomes).

## Parental Perinatal Mental Disorder and Infant Outcomes

### Behavioral Pathways: The Relationship Between Parental Perinatal Mental Disorder and Early Indices of Infant Psychobehavioral Functioning

Infants’ socio-emotional development is dynamically shaped throughout the first year as a result of their exposure to emotional expressions in everyday interactions. Indices of psycho-behavioral functioning at this period therefore focus on infants’ interactive behavior with their caregiver. Mental illness in parents in the first postnatal year seems to alter parents’ behavior in terms of affect expressions, attention, and sensitivity during these early interactions.

#### Parental Mental Illness and Parents’ Behavior and Affect in Early Interactions

Psychopathology in parents may interfere with parents’ experience and perceptions of their infant and alter parents’ behavior in everyday interactions with their child. Depressed and anxious mothers were observed to be less responsive and/or less sensitive to child signals than mothers without depression or anxiety during early interactions (69–72). Depressed mothers also display more neutral and negative, and less positive affect during their interactions with their infant (73). Moreover, evidence suggests that depression in parents is related to suboptimal amounts of stimulation in everyday activities; for example, depressed parents less often read, sing to, or play with their infants (72). Differently from depressed parents, anxious parents do not differ from reference parents in their positive or negative facial expressions during early interactions (74). Anxious parents, in turn, were reported to display “exaggerated behavior” which is defined by high intensity and frequency of gaze, facial expressions, and vocalizations that are inappropriate with regard to timing and content (75). Moreover, parents with diagnoses of social anxiety were found to show more anxious behavior during their interactions with a stranger in the presence of their infants (76, 77), while parents with panic disorder reported expressing more anger to their infants in disciplinary contexts (78).

The differences in parents’ emotional expressions and sensitivity are at least partly explained by psychopathology-related changes in parents’ perceptions of their child: For example, parents with depression were found to perceive their child as more negative (79) and to be less likely to detect happy facial expressions of their infants than parents without depression (80). Concerning parenting, depressed mothers’ behavior to their infant was classified as intrusive and overcontrolling on one end and withdrawn and understimulating on the other end of the continuum (81, 82). Withdrawn-depressed parents with depression were described to be less engaged and less tuned-in to their child during everyday interactions. Intrusive-depressed parents, in turn, seem to exert more control during play and intervene more frequently with their child’s exploration of novel stimuli (82). The withdrawn-depressed parenting style has been linked with an underresponsive physiological profile that is characterized by lower dopamine levels and higher right-frontal EEG activity than the intrusive-depressed style (83–85). These differences were proposed to reflect the behavioral inhibition (BI) and activation systems (83). On a parallel vein, the history of maltreatment in parents seems to indirectly contribute to nonoptimal patterns of parenting, which manifests as more negative and intrusive, as well as harsher parenting practices and less parental emotional availability (86–90). Thus, parents’ earlier negative experiences may at least partially explain the observed relationship between parents’ depression and parents’ negative perceptions of their child, and parenting practices (91).

Earlier evidence has also revealed a relationship between generalized anxiety symptoms and a more intrusive parenting style in parents with infants, along with less challenging parenting (92). Decreased levels of challenging parenting in anxious parents were proposed to be related to anxious parents’ reduced ability to encourage their child’s approach/exploration of potentially unsafe situations and to the development of child anxiety (93, 94). Findings from few studies that investigated parental behavior in early parent-infant interactions in parents with more severe mental disorders such as schizophrenia revealed that psychopathology-related alterations in mothers’ early interactive behavior are especially pervasive in the case of severe mental illness. For example, mothers with schizophrenia were found to be less sensitive, less responsive, and more withdrawn to their infant as compared to parents with affective disorders (95, 96). The effect of these psychopathology-related alterations in parents’ experience, perception, and responses to their child is suggested to be especially pronounced in the first postnatal year (48, 97).

#### Parental Mental Illness and Infant Expression and Regulation of Emotions in Early Face-to-Face Interactions

Psychopathology-related changes in their behavior and affect in early interactions may hamper parents’ ability to provide the optimal affective environment for infants’ emotional development. Theories of early socio-emotional development assign an important role to parents’ emotional expressions and regulation of emotions, as well as to affective synchrony (98, 99). Infants were shown to be highly sensitive to parental affective input at the first postnatal year: Studies in community samples reveal that they tune in to the subtle differences between their mothers’ and fathers’ expressions of affect in these interactions (100). Although infants have some primitive abilities to regulate negative arousal such as looking away or thumb sucking, these are highly reflexive and limited in effectiveness (101, 102). For the rest, infants highly rely on the assistance of their parents for regulating emotional experiences in negatively arousing situations. Co-regulation of infants’ emotional states in early dyadic experiences was suggested to lay the ground for the development of more voluntary emotion regulation strategies that emerge later in the first year (103).

Just like their parents, infants of depressed parents were shown to display more neutral and negative, and less positive affect than infants of reference parents during their interactions (73, 74, 104, 105) and to implement less mature emotion regulation strategies than infants of reference parents (106). Moreover, negative interactive style of depressed parents was suggested to trigger avoidance as an emotion regulation strategy: Children seem to use turning and gazing away from the mother as a strategy to regulate negative arousal possibly resulting from depressed parents’ limited sensitivity and responsivity (107). In line with this, it was found that infants of depressed parents use gaze aversion more often during their face-to-face interactions with their parents (108). Although avoidance can be seen as an adaptive strategy in response to parental depression as it would reduce infants’ exposure to parents’ negative affect, it may be less adaptive in other situations where it may restrict child’s exploration and new learning opportunities. On a parallel vein, it was suggested that due to their flat affect, limited responsibility and availability in everyday interactions, infants are less likely to actively seek input from depressed parents in ambiguous situations (109, 110).

Infants of anxious parents, in turn, more often display positive or negative expressions as compared to infants of reference parents in their face-to-face interactions with the parent (73, 111). The evidence also reveals that infants of anxious parents may express less negative affect as compared to infants of reference parents in challenging situations like meeting a stranger (75) but that they become anxious if they are first exposed to parental anxious displays before confronting the strangers (76, 77). In contrast, emotion regulation strategies of the infants of anxious parents do not seem to differ from infants of reference parents (106–108). In an earlier review on the links between exposure to parental depression and anxiety in the first postnatal year and child expressions of affect, it was suggested that infants’ displays of affect in everyday interactions in the case of parental depression and anxiety may be mirroring their parents (105): Infants who are repeatedly exposed to parents’ flat and negative affect in early face-to-face interactions may show a depressed interaction style characterized by more flat and more negative expressions. Similarly, infants exposed to parents’ anxious behavior in specific anxiety-provoking situations seem to show an anxious response characterized by avoidant tendencies in these situations as a result of modeling (76). Likewise, impairments in the parent-child early dyadic regulation of affect and the resulting difficulties in emotion regulation may constitute vulnerability for the development of psychopathology in children, especially in the presence of other vulnerabilities such as insecure attachment and difficult temperament.

#### Parental Mental Illness and Infant Attachment

According to attachment theory, neonates are biologically programmed to form a strong bond to their primary caregivers to ensure their survival (111). Parents’ ability to provide a timely and appropriate response to the infants’ dynamically changing attention and affective signals in everyday interactions at this period is of paramount importance for establishing a secure parent-child attachment in the early years of life (112, 113). Along with responsivity and sensitivity, parents’ mutuality and synchrony and their positive and supportive attitude during early interactions seem to be factors supporting the establishment of a secure attachment (111). It was suggested that early attachment in infants’ first relationships with the caregivers shapes one’s internal representations of relating to others. Attachment patterns show moderate stability from infancy to early adulthood years (114). Thus, although there is some room for change, infants’ attachment security in their early relationships with the parent provides the ground for later attachment behavior in personal relationships.

Infant attachment is commonly measured using the experimental paradigm the Strange Situation, which is a stressful situation involving parental separation and reunion, as well as stranger anxiety (115). The Strange Situation consists of a series of phases during which the parent leaves the child (alone or with a stranger) for a few minutes (parental separation) before she comes back and reunites with the infant (parental reunion). Several dimensions of infants’ behavior are observed during the reunion phase for measuring the attachment to caregiver, including infants’ proximity/comfort seeking versus avoidance, resistance against mothers’ attempt to contact and comfort them, and their emotional expressions. Securely attached infants express distress in response to maternal separation and positively embrace the reunion, while infants with resistant attachment experience stronger levels of stress in response to separation and show conflictual reactions to parental reunion, characterized by an approach to the parent for comfort, along with a resistance against it. In turn, infants with an avoidant attachment style do not seem to be distressed by maternal separation and/or interested to engage with the mother during the reunion.

A third pattern of insecure attachment, so-called disorganized/disoriented attachment, was later defined by Main and Solomon (116). Children with disorganized attachment overtly show disoriented/disorganized reactions to maternal separation and reunion episodes in the Strange Situation. These children show not only contradictory behavior (such as approaching the parent while averting gaze) and apprehension to the caregiver but also uncommon and out-of-context behavior such as freezing, sudden change in affect, fearful reactions to caregiver, and/or incomplete movements or atypical postures (117). Infants with disorganized attachment were suggested to seek contact with the primary caregiver, without a consistent or coherent strategy to establish that contact (116). It was suggested that at the core of the disorganized attachment style is a difficulty to trust and rely on parents for comfort and soothing. This may potentially be a result of repeated exposure to insensitive or disruptive parenting behavior (including frightening or frightened parental reactions) that is ineffective at meeting infants’ needs for proximity and comfort in stressful situations (118).

Earlier evidence has revealed that these insensitive and disruptive parenting behaviors may occur as a result of unresolved traumatic experiences including parents’ history of childhood maltreatment. In fact, more than half of the parents of infants with disorganized attachment were shown to have such unresolved trauma (119). In the case of childhood maltreatment, the links between earlier maternal trauma and security of parent-child attachment seem to be mediated by postnatal maternal depression (120). Infants’ exposure to parents’ postnatal depression and stress during early interactions seems to be linked to a lower likelihood of a secure attachment, along with a higher risk for insecure attachment (121–123). Moreover, higher rates of disorganized attachment were reported in the infants of mothers with borderline personality disorder (124). It is important to note that the association between parental mental illness and child attachment is rather modest in size and was not replicated in some of the more recent studies [for example, the link between parental psychopathology and disorganized attachment was not significant in the case of depression (125, 126), and in the case of anxiety (127, 128)]. Note, however, that most of the presented findings from these earlier studies are from community samples, whereas the association between parental mental illness and disorganized attachment would be especially pronounced in clinical samples of parents [for a more elaborate discussion, see Ref. (129)]. Although limited by similar methodological issues, a significant relationship between early insecure attachment and the development of internalizing and externalizing psychopathology from early childhood to adulthood years was reported in earlier studies (130, 131). To summarize, there is preliminary support for the idea that psychopathology-related alterations in parents’ behavior may be related to higher levels of insecure attachment in the offspring, which constitutes a vulnerability for intergenerational transmission of psychopathology. Further evidence from clinical samples of parents with infants is needed to reach firm conclusions about this link between parental psychopathology and insecure attachment.

#### Section Summary and Conclusions

Taken together, the evidence summarized in this section reveals a significant link between parental mental illness and parents’ parenting behaviors, and their expression and regulation of affect during early interactions. These psychopathology-related alterations may limit parents’ emotional availability and their ability to respond to their infant in a sensitive manner, rendering the early socio-emotional environment suboptimal for the establishment of a secure attachment bond, as well as for infants’ emotional development. Available evidence from infants of parents with anxiety and depression reveal that infants’ behavior during these early interactions, defined by high levels of affective negativity and avoidance, along with less mature emotion regulation skills, is reminiscent of the interaction and responses characterizing parents’ psychopathology. On the behavioral level, it seems that parents may already pass on negative interaction patterns characterizing affective psychopathology during these early interactions.

Long-term implications of the early suboptimal environment linked to perinatal parental mental health problems include a negative-insecure relational pattern that may be internalized and generalized to the offspring’s new relationships with teachers, peers, and romantic partners. The offspring may additionally face the risk of repeating early suboptimal relational experiences by choosing mentors, friends, and partners who behave in similar ways as the parent with psychopathology. Finally, the offspring of parents with perinatal mental disorders may adopt less functional emotional regulation strategies such as self-destructive behaviors, aggression, depression, or avoidance and may experience more difficulty regulating their negative emotions.

### Biological Pathways: The Links Between Parental Prenatal Mental Disorder and Early Indices of Infant Psychobiological Functioning

The first environment that a human being experiences is inside the mother’s womb. Research in the last decades has shown that this environment can have a great impact on the development of the embryo and fetus (132–135). The fetal programming hypothesis (136, 137) postulates that the environment of the developing fetus affects its development to enhance survival and prepares the infant for the environment to expect after birth. In the context of parental mental health, the mental state of the mother during pregnancy may influence the prenatal as well as the postnatal environment of the unborn child, thereby affecting its development. In this section, we discuss some of the possible mechanisms by which prenatal parental mental health may influence the development of the unborn child, with a focus on infant psychobiological development. We will mostly focus on maternal mental health during pregnancy with the womb as the first (biological) environment, even though fathers may directly and indirectly influence the environment of mother, and thereby her offspring. Furthermore, as mental illnesses co-occur with high levels of stress, and most research in this field is conducted on prenatal depression and anxiety, this section will focus on consequences of (traumatic) stress, depression, and anxiety during the prenatal period.

Human studies have shown that stress during pregnancy has widespread associations with offspring cognitive, emotional, and health outcomes (132–135). Studies in this area differentiate between different types of stress. That is, some studies investigate the impact of traumatic stressors that have happened during the prenatal period and that can be relatively objectively identified, such as having been exposed to the holocaust, the 9/11 attacks (138, 139), and natural disasters (140). Alternatively, some studies investigate the levels of stress that are subjectively experienced during pregnancy, either due to impactful events as mentioned above (141), due to daily life hassles, or due to the pregnancy itself (142, 143). Yet other studies examine more trait- or disorder-related experiences of stress, anxiety and depression (144). In this regard, studies in women that have developed or suffered from posttraumatic stress disorder or depression during the prenatal period often also focus on changes in stress physiology that are associated with these disorders in mothers (138, 145). Irrespective of the type of stress, most of the studies on prenatal stress indicate worse developmental outcomes with problems in the cognitive domain, emotional reactivity, and worse physical health outcomes. In this section we will discuss possible routes *via* which this psychobiological functioning of the infant can be affected by prenatal stress.

As human studies lack the possibility of randomly assigning stress during pregnancy to assess its impact, it is bound by the constraints of observational designs, and views differ on the origins of prenatal stress effects (137). However, studies that examine traumatic events that happened to a large group of people, such as a natural disaster, have the opportunity to more objectively compare women that have and have not suffered from these stressors. Animal studies on the other hand use experimental procedures, ranging from physical constraint to overcrowding, to induce prenatal stress (146). These studies are able to more directly examine causal effects of prenatal stress, independent of predisposing heritable characteristics or postnatal care, and give the opportunity to more precisely examine the potential underlying mechanisms by which prenatal stress may affect the prenatal environment of the fetus. Both human and animal studies comparing pregnancies with high levels of stress versus those with low levels of stress have given us insights in the psychobiological effects of prenatal stress and anxiety, some of which will be discussed next.

### The Links of Parental Mental Illness to Infant Psychobiological Development

Recent studies show that prenatal stress and mental health problems in mothers are associated with differential brain development in children (147), although studies in young infants are still rare (148). Some first studies in infants show associations between maternal prenatal depression and amygdala microstructure and functional connectivity in early infancy (149–151) and between maternal prenatal stress and amygdala functional connectivity in preterm neonates (152). Maternal prenatal anxiety has also been found to associate with infant brain microstructures and hippocampal growth (150, 151). Studies in rats complement these studies by showing that these effects can have a causative origin. Indeed, using restraint stress procedures or corticosterone administration in rats has been shown to affect brain morphology and behavior (146, 152).

One line of reasoning is that many of the effects of prenatal stress, anxiety, and depression on infant functioning and brain development are related to changes in the development of the infant HPA axis (153). The HPA axis plays a role in biological stress regulation, where brain areas like the hippocampus and prefrontal cortex are key brain areas regulating these stress responses, and is implicated in cognitive and emotional functioning (154). Quite a few human and animal studies show dysregulations in the HPA axis in relation to prenatal stress (46, 47). Both hyporeactivity and hyperreactivity of the HPA axis have been found in response to prenatal stress, and the effects seem to depend on timing and the type of the stress during pregnancy, time and type of HPA axis measurements, and child sex. For example, we showed that maternal prenatal anxiety was associated with heightened cortisol reactivity to a bathing session at 2 weeks of age but decreased cortisol reactivity to a vaccination at 2 months of age (142), showing moderation by time and type of stress induction. Brennan et al. (155) revealed that maternal prenatal depression was associated with increased baseline infant cortisol levels, while comorbidity with anxiety disorder was related to higher infant cortisol reactivity, showing differential effects on infant outcomes dependent on maternal disorder-specific symptoms. There are furthermore indications that females may be more susceptible to the impact of prenatal stress on HPA axis regulation (46).

Overall, the literature suggests that the HPA axis may be a key player in the association between prenatal stress and developmental outcomes, but longitudinal human studies showing proof for this pathway are still limited (156). From an evolutionary perspective, and according to the fetal programming hypotheses, prenatal stress would prepare the offspring for a stressful, dangerous, or hostile environment to grow up in. Changes in infant HPA axis regulation would thereby prepare for this environment. However, in case the postnatal environment is different than may be expected based on the first experiences, this can lead to a so-called mismatch in environments (157), in which the prenatal developmental changes do not lead to higher changes of survival but may induce susceptibility to pathology (47). While fetal programming has become an important area of research (136), the underlying mechanisms implicated in fetal programming still remain to be fully elucidated, and at different stages during pregnancy different mechanisms may play a role.

#### A Potential Mechanism: Prenatal Stress Hormones

One area that has been studied extensively in the context of prenatal stress, anxiety, and depression is the influence of maternal stress hormones, most notably cortisol, on the developing fetus. Maternal cortisol levels can directly influence fetal cortisol levels *via* the placenta or *via* stimulation of the infant HPA axis by placental corticotropin-releasing hormones (158, 159). While the fetus is in principle protected from high maternal cortisol concentrations by the placental enzyme 11β-hydroxysteroid dehydrogenase-type 2 (11β-HSD2), this enzyme is found to be inhibited by prenatal anxiety (160), reducing its protection against maternal cortisol. Heightened levels of cortisol during fetal development may in turn affect infant HPA axis regulation and brain development (161, 162). Besides changes in stress hormones, maternal prenatal stress or mental health problems may affect the unborn child in several other ways, including changes in inflammatory and metabolic conditions of the intrauterine environment (163). These endocrinological changes may be dependent on lifestyle factors (e.g., exercise, sleep, and nutrition) that could be direct consequences of heightened levels of stress, anxiety, or depression in the mother (132).

While the prenatal environment may be affected in many ways by changes in maternal hormones, and immune and/or metabolic status, in recent years the focus has shifted to underlying epigenetic mechanisms that may ultimately explain changes in the development of the fetus (135, 163, 164). Epigenetics refers to modifications to the genome that have functional consequences for gene functionality, without changing nucleotide sequences (165). The most common studied epigenetic factor in human research is DNA methylation, which is sensitive to glucocorticoid signaling (166). Epigenetic changes due to cortisol provide a route by which the prenatal environment can impact fetal development, as epigenetic changes due to prenatal stress hormones can directly impact gene activity and functionality during development of the fetal brain and HPA axis (167, 168). Interestingly, not only maternal stress but also paternal prenatal stress has been studied in this context. While paternal stress may impact maternal stress levels *via* behavioral and social routes, it has been suggested that stress in males can also lead to epigenetic changes in the sperm that can be directly transmitted to the offspring (169).

As discussed above, prenatal stress, anxiety, and depression affect the intrauterine environment and thereby the development of the fetus. However, these factors do not act alone and may interact with, or even represent, underlying genetic characteristics. First of all, the effects of maternal stress and mood can interact with genetic susceptibility of the unborn child (170). For example, child brain-derived neurotrophic factor (BDNF) genotype was found to moderate effects of maternal prenatal anxiety on later child internalizing problem behavior (171), as well as on the child’s epigenome and structures of the amygdala and the hippocampus (172). Secondly, an infant’s genetic susceptibility to emotional or developmental problems will depend on the genes of the parents. In that regard, associations between maternal and/or paternal stress, anxiety, and depression and infant development may partly be due to inherited characteristics (173). As such, dysregulations in the HPA axis of children may very well be directly inherited from the mother, possibly confounding previously discussed associations with prenatal stress. Similarly, the emotional development of children may depend on parental mental health *via* genetic routes. An interesting study by Rice at al. (173) has tried to disentangle some of these effects by comparing children that were born *via in vitro* fertilization (IVF), who were genetically either related or unrelated to the mother. They showed that prenatal stress affected birth outcomes and antisocial behavior independent of mother-child genetic relatedness, indicating prenatal stress as an environmental factor. Likewise, maternal anxiety and depression related to offspring anxiety levels held independent of relatedness. However, associations with symptoms of attention deficit hyperactivity disorder were only present in related pairs and hence implies underlying heritable factors (173). Such clever designs can give a more clear understanding of cause and effect when examining associations between prenatal or postnatal stress and infant outcomes.

So far, we have focused on mechanisms during the pregnancy. Obviously, prenatal stress may also be associated with changes in postnatal care, e.g., with regard to sensitive behavior or emotional availability, and hence affect infant development as well (132, 174); see the section Behavioral Pathways: The Relationship Between Parental Perinatal Mental Disorder and Early Indices of Infant Psychobehavioral Functioning. Furthermore, prenatal and postnatal mood disruptions in mothers can interact or have additive effects on child outcomes (137, 175, 176). In human studies, it is again hard to disentangle effects of the prenatal and postnatal environment, as each may have a different or continuous impact or reflect more underlying characteristics. Here as well, animal studies can guide in disentangling these environments by experimentally manipulating either prenatal or postnatal environment, and by cross-fostering studies (177).

#### Section Summary and Conclusions

In this section we show the importance of the first biological environment that the offspring experiences, i.e., the womb. Mothers’ prenatal stress and mental health status will influence the amount and diversity of hormones and metabolites that permeate the placenta and can thereby directly impact the development of the infant brain and physiology. These changes may be long lasting due to epigenetic changes that can permanently alter the phenotypic expressions of the infant, including heightened stress sensitivity and changes in HPA axis regulation. The long-term implications of these early alterations in infant psychophysiologic and biological functioning may go beyond heightened stress sensitivity and subsequent risk for mental disorders (e.g., anxiety, depression) as it also alters immunity and the brain-gut axis underpinning risk for somatic disorders (e.g., autoimmune diseases) later in development. However, it is important to note that these underlying mechanistic explanations need translational research in animals, as observational designs in humans limit our abilities to draw conclusions regarding the causality of observed associations between changes in parental and offspring psychobiology.

### Neurophysiological Pathways: The Links Between Parental Perinatal Mental Disorder and Early Neurophysiological Indices of Infant Psychological Functioning

An accumulating body of evidence illustrates that infants of mothers with mental illness are more likely to develop dysregulated behavior, lower levels of positive affect/behavior, and higher levels of externalizing and internalizing behavior (178, 179). From a developmental psychopathology perspective, child externalizing and internalizing behavior can be partly explained by individuals’ inability to regulate their emotions appropriately (180). Two physiological and neural indices play an important role in individuals’ emotion functioning. One is vagal tone, indexed by the respiratory sinus arrhythmia (RSA). Vagal activity is related to individuals’ facial expressions and to the process of physiological regulation during social engagement (181, 182). The second neural index is related to the amygdala: An enlarged amygdala or heighted connectivity between amygdala and other brain structures is related to heightened negative emotionality and affective disorders (151, 183, 184). In this section of the review, the focus is on the links between maternal mental illness and child’s physiological functioning as indexed by RSA and amygdala structure or amygdala connectivity.

#### Parental Mental Illness and Infant RSA

One of the underlying mechanisms explaining parent-to-offspring transmission of maternal depression and anxiety may be related to the activity in the parasympathetic system (178, 179). Recent evidence from experimental and correlational studies supports this idea (185–187). Activities in the parasympathetic system are usually indexed by vagal tone. The vagus nerve is part of the motor pathway that is connected to striated facial muscles that are responsible for social gaze, facial expression, and vocalization, supporting successful social engagement (182). RSA has been used to measure the functional output of the vagal pathway on the heart (190). It refers to the variability in heart rate that occurs at the frequency of spontaneous respiration. Higher baseline RSA is an index of flexible responding (191) and is linked to better self-regulation (192) and better sustained and focused attention (188, 189). However, higher baseline RSA is also found to be related to greater behavioral reactivity (193) and heightened frustration (192).

The prenatal period and the first year of life are critical periods for the maturation of the vagal system (182, 194), which is indexed by the number of myelinated vagal fibers. Without a working myelinated vagus, more rudimentary defensive strategies such as fight-flight mobilization, tantrum, and shutdown behavior will dominate rather than regulate social behaviors (182). The myelinated vagal fibers keep burgeoning in number, and the myelin thickness continues to increase from 24 weeks through adolescence; however, the greatest increase is observed from 30–32 weeks of gestational age to approximately 6–9 months postpartum (195, 196). Thus, maternal psychopathology [for example, maternal depression reflected in flat affect, unresponsiveness, and low sensitivity (197)] may exert a stronger effect during this stage than later in development.

Infants of mothers who experience prenatal or postnatal depression were shown to be more likely to exhibit lower baseline RSA as early as neonates (84, 198). Infants of mothers with postnatal depression also do not show the usual increase in RSA that is observed from 3 to 6 months in typical development (198). Similar findings were reported in infants of mothers with anxiety disorders (either during lifetime or during pregnancy (199, 200). Low baseline RSA poses several disadvantages for infants (181). Given its connection to the striated facial muscles, the nonoptimal vagal development may impede infants’ ability to signal or express their emotions, which in turn may increase infants’ risk of developing affective disorders (181, 201). Observational studies support this view such that newborns of depressed (versus nondepressed) mothers showed fewer facial expressions in response to happy and surprised facial expressions (202) (also see the section Behavioral Pathways: The Relationship Between Parental Perinatal Mental Disorder and Early Indices of Infant Psychobehavioral Functioning). Moreover, lower baseline RSA levels limit infants’ ability to engage in physiological regulation (203). Taken together, evidence generally supports the idea that infants who have depressed and/or anxious mothers may have difficulty expressing emotions resulting from their nonoptimal development of RSA, and this may in turn impede their social engagement, enhancing the risk for later development of depression and anxiety.

Opposite to lower baseline RSA in infants that is generally seen as maladaptive (181), high baseline RSA is defined as a “biological sensitivity to context” factor (204, 205) such that infants with higher RSA are more susceptible to the environmental influences for better and for worse. This idea is supported by recent evidence that revealed that maternal depression and anxiety are linked to maladaptive infant outcomes (e.g., infant negativity, sleep problems, or disorganized attachment) only for infants who showed higher baseline RSA but not for infants who showed lower baseline RSA (206–208). Thus, in the context of parental mental illness, the finding that infants with higher baseline RSA demonstrate more maladaptive outcomes possibly indicates a misfit between infants’ physiology and the level of stress in the environment. Further studies are needed to elucidate the effect of baseline RSA servicing as a “biological sensitivity to context” factor (205).

#### Maternal Mental Illness and Infant RSA Withdrawal

Differently from the Baseline RSA that is usually seen as an index of a stable resting “physiological state” (181, 203), a decrease in RSA or RSA withdrawal reflects individuals mobilizing resources in response to immediate environmental challenges, such as dealing with a frustrating or stressful situation. This process facilitates an increase in heart rate and allows individuals to shift from maintaining internal homeostasis to coping with external demands (201). After the stressor is over, individuals usually experience a recovery that manifests an increase in RSA (201). Consistent with the theory, the process of infants’ RSA withdrawal is associated with concurrent behavioral regulation and recovery from distress (190, 209). A meta-analysis has revealed that children who were able to engage in RSA withdrawal during stressful situations had fewer externalizing, internalizing, and cognitive/academic problems; moreover, lower levels of RSA withdrawal were found in children who displayed clinically elevated behavior problems (210).

Young children have limited ability regulating their negative arousal, and the caregiver serves as an important external regulator for infants *via* physical contact and verbal confirmation (211). Parents who engage in sensitive and responsive parenting usually have infants engaging in optimal levels of RSA withdrawal and normative RSA recovery (212, 213). However, for parents who experience mood disorders, the dyadic coregulation process is likely to be disrupted considering that the mothers’ fatigue and depressed mood may result in inability to respond to the infants’ need in a timely and sensitive manner (104, 197, 214). Thus, infants lose the opportunities of learning to down-regulate their negative arousal, and they are more likely to develop physiological dysregulation in the long run (211). Empirical studies that considered multiple risk factors in mothers showed that infants in the high-risk group (characterized by mothers’ current mental disorder, substance use, or two or more psychosocial risk factors) showed no recovery during the reunion episode of the Still-Face Paradigm suggesting a dysregulated physiological response in infants (187). In another study, no difference was reported in RSA changes between infants of mothers with depression and the control group (215). In contrast, infants whose mothers had bipolar disorder were shown to exhibit an increase in RSA during the stressor task compared to the control group in this study, indicating nonoptimal physiological regulation during a stressful task. To sum up, there is some indirect evidence (i.e., the effect of mood disorder is not teased out) that infants of mothers with mental illness, especially mood disorders, are more likely to develop physiological dysregulation (187, 215). However, more research is needed to uncover the direct association between parental mental illness and infant physiological regulation. Finally, note that no evidence is yet available on the links between paternal mental disorders and infants’ vagal functioning. Considering that fathers’ mental illness exerts its influence on the children either directly through parenting behaviors or indirectly through negatively affecting mothers’ parenting behaviors (216–218), resulting in nonoptimal development in infants’ physiological functioning, it is important to incorporate fathers into future studies on this line of research.

#### Maternal Mental Illness and Amygdala Activity in Infants

The amygdala, a critical brain region in the processing of threat, is susceptible to environmental adversity in early development (219). Mothers with prenatal depression are likely to experience multiple changes physiologically that may affect fetal development such as an increased cortisol production (220, 221). The amygdala is one of the areas rich in glucocorticoid receptors in the fetus’ brain, which seems to be especially negatively affected by maternal cortisol levels (222). Increased amygdala activation in response to novelty or threat in children has been linked to higher negative emotionality (223). Furthermore, a larger amygdala in volume, strengthened amygdala connectivity, and greater right amygdala activation are all associated with an increased risk of developing affective disorders such as depression in children and adolescents (183, 184, 224, 225).

Evidence reveals prenatal depression may have a significant effect on the differences in the microstructure of the right amygdala in neonates after controlling for postnatal depression (151). More specifically, significantly lower anisotropy and axial diffusivity, which contribute to increased negative emotionality, were observed in neonates of prenatally depressed mothers (151). Furthermore, evidence supports the idea that maternal depression may also alter the amygdala connectivity in infants. Prenatal depression was shown to be linked to greater functional connectivity in the amygdala with the left temporal cortex and insula, as well as the bilateral anterior cingulated, medial orbitofrontal, and ventromedial prefrontal cortices in 6-month-old infants; these patterns are correlates of major depressive disorder in adolescents and adults (150). Therefore, the changes in the amygdala structure and amygdala connectivity may increase infants’ vulnerability of developing affective disorders and may serve as another important mechanism through which prenatal mental illness, specifically depression, is transmitted to infants (151, 226).

#### Section Summary and Conclusions

Physiological and neural indices serve as underlying mechanisms that may be involved in the transmission from prenatal mental illness to infants’ maladaptive functioning. Evidence from literature examining RSA and amygdala activity illustrates that infants of parents with mental illness are more likely to carry physiological risk factors such as lower baseline RSA, reduced RSA withdrawal, and heightened amygdala connectivity. In the long term, these early alterations in RSA and amygdala connectivity may, through mechanisms such as difficulties in emotional expressions, emotion regulation and threat sensitivity, may increase infants’ vulnerability of developing mental disorders such as depression and anxiety disorders. Further research on moderating influences (e.g., children’s resilience factors and parenting behavior) of the link between parental mental illness and infant physiological and neural functioning is needed before drawing conclusions on responsible mechanisms.

## Effect of Early Interventions on Parent and Infant Outcomes

The findings summarized in earlier sections illustrate the potential value of early interventions targeting parents’ psychopathology and related alterations in early parent-infant interactions in the prevention of intergenerational transmission. In light of the short-term and longer-term risks associated with parental perinatal psychopathology [e.g., Refs. (52, 56, 106, 227)] interventions for parents experiencing perinatal psychopathology have focused on infant as well as parent treatment outcomes.

Here, we provide an overview of the interventions for parents with a diagnosed psychiatric disorder [so not, for example, the interventions such as (228–231), where mothers were not diagnosed with psychiatric disorders and where the intervention began before 12 months [so not, for example, Ref. (232) or (233)].

Research into interventions for parents experiencing perinatal psychiatric disorders has predominantly focused on depression, with very few exceptions [for example, a trial for mothers with bulimic eating disorders (234), a trial for mothers with postpartum OCD (235), and a trial registered, but not yet reported, for mothers with anxiety disorders during pregnancy (236); for systematic reviews and meta-analyses, see for example Refs. (237, 238)]. We focus primarily on interventions examined in randomized controlled trials (RCTs), and then only briefly address the interventions examined using less robust designs.

We must emphasize that, to our knowledge, no intervention study has focused on paternal mental disorders and infant outcomes. For over a decade, research has addressed the risks posed by paternal psychopathology (59). It appears that risk pathways from paternal postnatal depression overlap with, but are not identical to, those of depressed mothers (239). Paternal anxiety disorder has received less attention, but, in infancy and toddlerhood, fathers’ social anxiety appears to be as important as mothers’ in predicting offspring anxiety (76, 240). So, while paternal psychopathology is important, evidence from trials addressing the effect of paternal interventions has yet to be reported.

### Interventions for Maternal Mental Illness

Postnatal depression has been the most frequently studied postnatal psychiatric disorder with respect to interventions to address infant outcomes. This section provides an overview of progress in the field, moving from trials examining infant outcomes where maternal postnatal depression alone was the focus of treatment, to trials where mother-infant interactions have been the treatment targets, to having *both* maternal postnatal depression *and* mother-infant interaction as the treatment targets [for systematic reviews for broader considerations (237, 238, 241)].

#### Maternal Postnatal Depression as the Intervention Target

Two RCTs have examined infant outcomes following treatment of maternal postnatal depression alone (242, 243). The first trial (242, 244) examined the effect of three treatments (psychodynamic psychotherapy, cognitive behavior therapy, and nondirective counseling) versus routine primary care on maternal and offspring outcomes up to 5 years. Although all three treatments were associated with improved depression symptoms compared to routine primary care at the end of treatment (18 weeks postpartum), prevalence of maternal depression diagnosis was reduced only in mothers who received brief psychodynamic psychotherapy. At 5-year follow-up, compared to routine primary care, the treatments had led to no reduction in episodes of depression (244). Regarding offspring outcomes at the end of treatment, mothers in all treatment groups reported lower levels of problems in their relationships with their offspring compared to mothers in routine primary care. Mothers facing high social adversity and receiving nondirective counseling also reported more maternal sensitivity. However, none of the interventions was associated with effects on child attachment or cognitive development compared to the control group, and no effects were found at 5 years on measures of child emotional, behavioral, and cognitive development.

The second RCT (243) tested whether improved maternal mood led to improved child outcomes. Depressed mothers were randomly allocated to either interpersonal psychotherapy (IPT, n = 60) or to a waitlist control group (n = 60), and 56 nondepressed mothers served as control group for comparison. At the end of treatment (mean average, 9 months postpartum), compared to the waitlist control, IPT was superior only in the domain of parenting stress (although this remained higher than in the nondepressed group). At 18 months postpartum, compared to the offspring of nondepressed control mothers, offspring of mothers who received treatment had more behavior problems, lower attachment security, and more negative temperament. In summary, these early RCTs suggested that treatment of maternal postnatal depression alone was inadequate to ameliorate the risk posed to offspring by maternal postnatal depression.

#### Mother–Infant Relationship as the Intervention Target

In light of results from interventions focused on maternal postnatal depression alone, two RCTs (245, 246) examined the effects of interventions in the context of maternal postnatal depression where the intervention target was the mother-infant relationship, not maternal postnatal depression. First, Van Doesum and colleagues (245) examined the effects of 8 to 10 sessions of home-based video feedback treatment (VFT) (n = 35) and a control treatment of three 15-min telephone sessions offering practical parenting advice (n = 36) on infant attachment and maternal sensitivity. The study did not include treatment for depression. Regarding effects on mothers’ behaviors, at the end of treatment and at 6 months follow-up, mothers in the VFT group were observed to be more sensitive and to provide more structure in their interactions with their infants compared to mothers in the control group. Regarding children’s development, at the end of treatment, children of mothers who received VFT were observed to be more responsive to their mothers and more involved in interactions when compared to offspring of mothers in the control group. At the 6 month follow-up, prevalence of secure attachment status were higher for offspring of mothers who received VFT. These results must be considered in light of possible attention effects of the intervention (8 to 10 home visits) compared to the control group (three 15-min telephone calls). At 5-year follow-up (247), no main effects of treatment were found for mothers or offspring. However, where families experienced stressful life events, children in the VFT group had fewer mother-reported child externalizing problems than children in the control group. Thus, these results suggested that early, intensive intervention focused on the mother-infant relationship could alter infant development in key domains. Moreover, for those facing further risk in light of subsequent stressful life events, possible protective effects were reported against child externalizing problems.

Second, Horowitz and colleagues (246) reported an RCT with 136 mother-infant dyads, where mothers received an intervention called Communicating and Relating Effectively (CARE) designed to teach mothers to identify, and respond sensitively to, their infant’s behavioral cues, or no treatment. All mothers were visited at home at 6 weeks, 3, 6, and 9 months postpartum for observational assessments, with the CARE group receiving additional visits at 2 and 4 months to receive the CARE intervention. Both groups improved on measures of maternal depression, mothers’ behaviors, and mother-infant interactions, but there were no significant differences between groups. It is possible that any effects of the two sessions of the CARE intervention were confounded by the attention given to the control group (that is, four home-based observational visits). Further, the mean baseline score on the Edinburgh Postnatal Depression Scale (EPDS) was under 13 for both groups, suggesting that the depression was insufficiently severe to lead to adverse child outcomes. To summarize, the VFT treatment examined by Van Doesum and colleagues (245, 247) reported promising effects for infants and, at 5-year follow-up, protective effects for children who had experienced more stressful life events. Horowitz and colleagues (246) in contrast found no effect of their CARE program. While the interventions in these two trials both focused on helping depressed mothers identify and respond sensitively to their infants’ cues, the different “doses” in the two studies, 10 sessions of VFT and two sessions of CARE, might account for the inconsistent results.

In summary, studies examining interventions with their target as *either* maternal depression (see the section Maternal Postnatal Depression as the Intervention Target) *or* the mother-infant relationship (see the section Mother–Infant Relationship as the Intervention Target) have yielded little evidence of short-term benefit to offspring development and almost no benefit at longer-term follow-up. Recent evidence points to the importance of the severity and the persistence of postnatal depression as moderators of risk for adverse childhood and adolescent development (227). In the intervention studies summarized above, the severity of maternal depression (for example, a mean score on the EPDS in the mild to moderate depression range) and the timing of interventions (being completed between 4.5 and 9 months postpartum) possibly limited these studies’ ability to clarify the effects of intervention on infant development.

#### Maternal Postnatal Depression and Mother–Infant Relationship as the Intervention Targets

The first study to examine children’s outcomes in the context of severe and persistent maternal postnatal depression, where the mother–infant relationship was a target while mothers also received an evidence-based treatment for depression, was reported by Stein and colleagues (248). In this RCT, 144 mothers were randomly allocated to receive, at home, either video feedback therapy (VFT, with the mother–infant relationship as its target; N = 72) or Progressive Muscle Relaxation (PMR, with stress management as its target; N = 72). Concurrently, all mothers received cognitive-behavioural therapy (CBT) for depression at home (10 sessions between 6 and 12 months postpartum, with two booster sessions in the second postnatal year). In particular, the study examined putative mediators of children’s development in the context of postnatal depression, by attempting to use VFT to modify key maternal behaviors (sensitivity, warmth, and contingent responsiveness) which have been shown to be a) impaired in the context of postnatal depression and b) associated with adverse child outcomes (in attachment, behavioral, and cognitive domains). Regarding mothers’ parenting behaviors, groups did not differ at the end of treatment or when children were 2 years old. Regarding children’s outcomes at 2 years, development was examined in the domains of attachment, behavior, and cognitive development. In all these domains, children’s development did not differ between the two groups but was found to be comparable with normative development in nonclinical samples. Stein and colleagues proposed that, given maternal depression had remitted in over 80% of mothers by the end of the first year, and over 85% by the end of the second year, children’s developmental outcomes could be understood in the context of no exposure to maternal depression from late in the first year through to the end of their second year. Thus, intensive treatment of maternal depression up to the end of the first year together with the interventions on mother-infant interactions could be adequate to mitigate the impact of maternal postnatal depression on children’s development at 2 years.

The trials reviewed above all addressed postnatal depression. The impact on infants of interventions for prenatal depression has received relatively little attention to date. Results are promising, with significant benefits for infants from two pilot RCTs. In their pilot RCT comparing individual, home-based CBT with treatment as usual (TAU) for ante-natal depression, Netsi and colleagues (249) found no significant differences in infant outcomes by treatment. Improved prenatal depression symptoms, however, were associated with easier infant temperament and shorter infant sleep duration 2 months postnatally. Milgrom and colleagues (250) found that group CBT for prenatal depression, compared to usual care, had medium to large effects on infant self-regulation, stress reactivity, and problem solving at 9 months old. These infant outcomes were obtained even when controlling for postnatal depression symptoms. While both pilot studies provide encouraging results, as pilot studies, neither was designed to examine hypotheses regarding fetal programming effects (173). Larger trials will be required to examine the mechanisms of *how* treatment of prenatal depression has its impact on infant development.

So far, we have only reviewed studies reporting RCTs that specifically focused on perinatal depression. However, there are other promising early intervention studies that depressed mothers may profit from and that are worth mentioning briefly. For example, in mindfulness-based programs, parents learn to relate differently to their own psychopathology and to their child (fostering more attentive and less overreactive parenting) through meditation practices. For example, Mindfulness-based Child birthing and Parenting (251, 252), an intervention for pregnant women and their partners, is found to reduce anxiety and depression in both the pregnant women and their partners (250) who play a role in buffering or increasing stress, anxiety, and depression of the future mother during pregnancy. Another intervention for mothers with psychopathology, Mindful with your baby, targets early parenting, babies with (regulation) problems, and mother-baby interaction problems (254, 255). Mindful with your baby was shown to lead to improvements in mothers’ psychopathology, babies’ or infants’ behavior problems, and mothers’ observed parenting and the mother-child interaction.

As the literature stands, in the context of maternal perinatal depression, short-term benefits in infant development have followed successful modification of maternal parenting behaviors, with benefits for children’s development evident at 5 years of age where children had experienced stressful life events. Conversely, the impact of persistent postnatal depression on children’s development can be mitigated, but *via* effective treatment of depression in the first postnatal year, sustained over the second year, without modification of the maternal parenting behaviors impaired by postnatal depression (PND).

Regarding mental illnesses other than depression, literature is less well developed. For example, for mothers with a range of mental illnesses, Fonagy and colleagues (231) conducted an RCT of Parent–Infant Psychotherapy (PIP), compared to TAU, for effects on infant cognitive, language, and motor development. When compared to TAU at 12 months, PIP had no effect on infant cognitive, language, or motor development. To enhance maternal parenting and infant outcomes in the context of maternal substance abuse disorders, Pajulo and colleagues (256, 257) have developed an intervention to promote maternal reflective functioning (RF). In a case series with 34 mother-infant pairs, they reported a significant increase in maternal RF from pretreatment to posttreatment, and that better RF was negatively associated with later relapse to substance use and children being placed in foster care (257). More robust research designs are required to establish the possible effects of enhancing maternal RF in the high-risk context of substance abuse disorders for infant outcomes.

### Section Summary and Conclusions

Presently, it appears that treatment of depression prenatally may have beneficial effects on infants’ self-regulation, stress reactivity, and temperament. However, postnatal interventions addressing either parental psychopathology or parent-infant relationship in isolation do not seem to significantly improve child outcomes. On the other hand, the combination of interventions targeting parental depression together with interventions on parent-infant relationship or with parental stress management show some promise in adequately limiting infants’ exposure to the disorder’s impact. It remains to be shown whether these positive effects extend beyond the end of the second postnatal year. Finally, the mechanisms *via* which positive infant outcomes can be achieved remain unclear. Research might fruitfully elucidate how interventions have their effects on enhancing children’s outcomes by targeting those who face risks in addition to parental perinatal psychiatric disorder. For example, in addition to parent anxiety disorders infant BI is a risk factor for Social Anxiety Disorder (258). Thus, examining whether the effects of intervention for postnatal parental anxiety differ according to infant temperament (BI or not BI) could show how an intervention impacts infants’ development [for example, *via* modifying one or both of postnatal anxiety disorder and BI (259)]. Effective early interventions targeting parental mental disorders and the parent-infant relationship may have a profound beneficial impact on the development of the child up to adulthood in many ways. Potentially such effects may even impact the next generation, as parenting experiences will affect future parenting behavior. As reflected in the focus of this intervention section, we require interventions for other psychiatric disorders and for fathers experiencing perinatal psychiatric disorders.

## Discussion

The current review provided a snapshot of the period between pregnancy and the first postnatal year among parents with mental disorders and their children by focusing first on the links between parental mental illness and behavioral, biological, and neurophysiological correlates of infant psychological functioning in this period. Next, to provide insight to the question of whether interventions may help to reduce or reverse this link, we focused on the effects of early interventions targeting parental mental illness (and/or) parenting on infants’ psychological outcomes. The summarized evidence provides preliminary support for the idea that parental psychopathology may limit parents’ ability to provide an optimal environment for the offspring’s emotional and physiological development in this sensitive period where parents’ synchrony, responsivity, affect expression, and regulation lays the necessary ground for healthy development in infants. The evidence further suggests that these psychopathology-related changes in parents’ behavior and biology in the perinatal period may be related to significant alterations in brain development and to behavioral, biological, physiological, and neural correlates of infant psychological functioning in this period. The accompanying changes in infants’ behavioral, biological, neural, and physiological profile seem to be reminiscent of the responses characterizing parents’ psychopathology. For example, infants of depressed parents express less emotion and engage less in positive interactions, show lower vagal tone, stronger right frontal EEG activation, and elevated cortisol levels. These altered profiles in themselves may constitute risk for later development of child and/or adult forms of psychopathology and thus for intergenerational transmission.

These findings highlight the essential value of early interventions to alleviate the transmission of psychopathology risk from mentally ill parents to their infant. Although targeting depression or mother-infant interactions in isolation may not be sufficient in the postnatal period, intensive interventions targeting depression earlier, i.e., prenatally, and or more intensively—along with mother-infant interactions—may be promising in alleviating the risk of early transmission. It is important to underline that these early infant psychological profiles that are related to parental mental illness summarized in this article are only probabilistically related to later development of psychopathology and may not fully account for the intergenerational transmission of psychopathology. In fact, not all children of mentally ill parents develop psychopathology or maladaptive outcomes. From a developmental psychopathology perspective, psychopathology in the offspring of mentally ill parents at a given point in development emerges as a result of complex and dynamic interactions between risk and resilience factors operating at the psychological, biological, and social levels of influence up to that point (260). Later adaptation/maladaptation of the offspring certainly depends on further adversity or opportunities that may either aggravate or alleviate the transmitted risk in early development (97, 260, 261). Finally, as child characteristics such as BI start to play an increasingly pronounced role from infancy onwards (262), the bidirectional nature of the associations between parent and child outcome is important to consider in familial transmission.

Although our focus was exclusively on parental mental illness as a risk factor for psychopathology in this review, the inherent complexity of multiple risk/resilience factors and mechanisms that dynamically operate in the development of psychopathology in the offspring makes it necessary to consider the influence of other factors along with parental mental illness and the interventions. These factors include more proximal influences related to the characteristics of the parent [such as history of childhood abuse (90, 91)], the child [such as temperament or BI (258, 262) and gender (140)], the couple [such as coparenting (263) and marital satisfaction (264)], and the more distal influences regarding the family and culture and broader socio-economic determinants. Future studies that incorporate these factors in longitudinal designs in mentally ill parents from pregnancy up to the point where child psychopathology develops will be essential for a more complete understanding of intergenerational transmission.

Moreover, it is important to evaluate the conclusions in view of the limitations coming from the scope of the parental mental disorders addressed by the evidence, as well as by the methodological limitations inherent to the study designs. The summarized evidence predominantly comes from depression, followed by anxiety and traumatic stress, whereas this is likely to change, now that there is an increased recognition of the fact that all disorders along the diagnostic spectrum may manifest during pregnancy and the postnatal period in mothers and fathers (4, 17–19). Methodologically speaking, the reported associations between parental mental illness and infant outcomes are from semi-experimental designs, which preclude any causal inferences. The longitudinal designs therefore provide a unique advantage in establishing a timeline between infants’ exposure to parental mental illness and the corresponding alterations in infant outcomes. Finally, methodological limitations are related to the chronic nature and continuity of parental psychopathology from the prenatal period onwards, which make it difficult to delineate the prenatal influence from postnatal and postnatal influence from later effects of psychopathology.

Finally, we note that, despite substantial psychopathology among (future) fathers, and taking into account that most children are raised by two parents, a mother and a father, most studies on the role of parental psychopathology and interventions focused on mothers, disregarding the various roles that parents play directly (for example, through exposure to paternal mental illness) and indirectly (for example, *via* buffering or increasing the psychopathology-related stress in the mother or in the triad). Future studies will need to elucidate these influences by including fathers or co-parents in their future research designs.

## Final Conclusion and Implications

The available evidence reviewed in the current study leaves no doubt about the importance of reaching men and women with a mental health problem who become parents or who are planning or expecting to become parents as early as possible. A recent meta-synthesis on the factors that prevent women with mental illness to reach out to healthcare services for support during the pregnancy and postnatal year provides insight to the potential ways of enhancing the use of healthcare services and reducing the isolation that mothers experience on the way to and/or in the early phases of parenthood (265). First, the stigma and fears about the loss of custody can be reduced *via* informing the general public on the broader scale and this specific group on a smaller scale about the high prevalence of mental illness in this period and about the possibilities of alleviating the effect of parental mental illness on the parent and the child. Second, it seems that providing some stability on who delivers the care and integrating the services such that the different components can be delivered by the same professionals who are open and accessible to share psychological needs may largely improve the experience of healthcare among individuals with mental illness. Third, a nonjudgmental and compassionate approach and a readiness to provide the needed information by health professionals have been highlighted as important qualities that may facilitate the help-seeking of men and women with mental illness for healthcare services in the perinatal period. Finally, putting an equal weight on the parents’ and the baby’s needs and involving the parents with mental health problems in the decision-making process related to medical and psychological treatment are of golden value in providing an optimal healthcare environment that parents with mental health problems may turn to whenever needed.

## Author Contributions

EA wrote the first drafts of the sections Introduction and Discussion, Final Conclusion and Implications and authored the section Behavioral Pathways: The Relationship between Prental Perinatal Mental Disorder and Early Indices of Infant Psychobehavioral Functioning. MT, JQ, and PL authored the sections: Biological Pathways: The Links Between Parental Prenatal Mental Disorder and Early Indices of Infant Psychobiological Functioning, Neurophysiological Pathways: The links Between Parental Perinatal Mental Disorder and Early Indices of Infant Neurophysiological Functioning, and Effect of Early Interventions on Parent and Infant Outcomes, respectively. BE and SB provided advice on the scope, structure, and content of the manuscript and contributed to the writing and revisions of the sections Introduction, Discussion and Final Conclusion and Implications. All authors contributed to manuscript revision and read and approved the submitted version.

## Funding

The contribution of EA was supported by the Dutch National Science Foundation (Rubicon grant number 446-16-021).

## Conflict of Interest Statement

The authors declare that the research was conducted in the absence of any commercial or financial relationships that could be construed as a potential conflict of interest.
